# The role of US examination in the management of acute abdomen

**DOI:** 10.1186/2036-7902-5-S1-S6

**Published:** 2013-07-15

**Authors:** Maria Antonietta Mazzei, Susanna Guerrini, Nevada Cioffi Squitieri, Lucio Cagini, Luca Macarini, Francesco Coppolino, Melchiore Giganti, Luca Volterrani

**Affiliations:** 1 Department of Medical, Surgical and Neuro Sciences, Section of Radiological Sciences, Siena, Italy; 2 University of Perugia, Thoracic Surgery Unit, Perugia, Italy; 3 University of Foggia, Department of Radiology, Foggia, Italy; 4 University of Palermo, Department of Radiology, Palermo, Italy; 5 University of Ferrara, Dipartimento di Scienze Chirurgiche, Ferrara, Italy

## Abstract

Acute abdomen is a medical emergency, in which there is sudden and severe pain in abdomen of recent onset with accompanying signs and symptoms that focus on an abdominal involvement. It can represent a wide spectrum of conditions, ranging from a benign and self-limiting disease to a surgical emergency. Nevertheless, only one quarter of patients who have previously been classified with an acute abdomen actually receive surgical treatment, so the clinical dilemma is if the patients need surgical treatment or not and, furthermore, in which cases the surgical option needs to be urgently adopted. Due to this reason a thorough and logical approach to the diagnosis of abdominal pain is necessary. Some Authors assert that the location of pain is a useful starting point and will guide a further evaluation. However some causes are more frequent in the paediatric population (like appendicitis or adenomesenteritis) or are strictly related to the gender (i.e. gynaechologic causes). It is also important to consider special populations such as the elderly or oncologic patients, who may present with atypical symptoms of a disease. These considerations also reflect a different diagnostic approach. Today, surely the integrated imaging, and in particular the use of multidetector Computed Tomography (MDCT) has revolutionised the clinical approach to this condition, simplyfing the diagnosis but burdening the radiologists with the problems related to the clinical management. However although CT emerging as a modality of choice for evaluation of the acute abdomen, ultrasonography (US) remains the primary imaging technique in the majority of cases, especially in young and female patients, when the limitation of the radiation exposure should be mandatory, limiting the use of CT in cases of nondiagnostic US and in all cases where there is a discrepancy between the clinical symptoms and negative imaging at US.

## Background

The term “acute abdomen” can be defined as a medical emergency, in which there is recently onset sudden and severe pain in the abdomen with accompanying signs and symptoms that focus on an abdominal involvement. Abdominal pain can be classified as visceral, somatoparietal or referred pain that can be a manifestation of a wide array of systemic and local causes. More common causes are cholecystitis, acute appendicitis, bowel obstruction, visceral perforation, mesenteric ischemia and ischemic colitys in elderly patients. However acute abdomen can represent a wide spectrum of conditions, ranging from a benign and self-limiting disease to a surgical emergency. Nevertheless, only one quarter of patients who have previously been classified with an acute abdomen actually receive surgical treatment, so the clinical dilemma is if the patients need surgical treatment or not and, furthermore, in which cases the surgical option needs to be urgently adopted [1,2]. Therefore, a thorough and logical approach to the diagnosis of abdominal pain is necessary. Some Authors assert that the location of pain is a useful starting point and will guide a further evaluation that the American College of Radiology has recommended using different imaging studies to assess abdominal pain based on pain location: ultrasonography (US) is recommended to assess the right upper quadrant pain, and computed tomography (CT) is recommended for the right and left lower quadrant pain (Table 1) [3]. However some causes are more frequent in the paediatric population (like appendicitis or adenomesenteritis) or are strictly related to the gender (i.e. gynaechologic causes). It is also important to consider special populations such as the elderly or oncologic patients, who may present with atypical symptoms of a disease. Due to these important implications, a practical classification of acute abdomen divides this condition into three different groups: children acute abdomen, female acute abdomen and male acute abdomen. This concept also reflects a different diagnostic approach.

**Table 1:** Differential Diagnosis of Abdominal Pain according to Pain location^1^

*Pain location*	*Possible diagnoses*
Right upper quadrant	Biliary: cholecystitis, cholelithiasis, cholangitis Colonic: colitis, diverticulitis Hepatic: abscess, hepatitis, mass Pulmonary: pneumonia, embolus Renal: nephrolithiasis, pyelonephritis
Epigastric	Biliary: cholecystitis, cholelithiasis, cholangitis Cardiac: myocardial infarction, pericarditis Gastric: esophagitis, gastritis, peptic ulcer Pancreatic: mass, pancreatitis
Left upper quadrant	Cardiac: angina, myocardial infarction, pericarditis Gastric: esophagitis, gastritis, peptic ulcer Pancreatic: mass, pancreatitis Renal: nephrolithiasis, pyelonephritis Vascular: aortic dissection, mesenteric ischemia
Periumbilical	Colonic: early appendicitis Gastric: esophagitis, gastritis, peptic ulcer, small-bowel mass or obstruction Vascular: aortic dissection, mesenteric ischemia
Right lower quadrant	Colonic: appendicitis, colitis, diverticulitis, IBD, IBS Gynecologic: ectopic pregnancy, fibroids, ovarian mass, torsion, PID Renal: nephrolithiasis, pyelonephritis
Suprapubic	Colonic: appendicitis, colitis, diverticulitis, IBD, IBS Gynecologic: ectopic pregnancy, fibroids, ovarian mass, torsion, PID Renal: cystitis, nephrolithiasis, pyelonephritis
Left lower quadrant	Colonic: colitis, diverticulitis, IBD, IBS Gynecologic: ectopic pregnancy, fibroids, ovarian mass, torsion, PID Renal: nephrolithiasis, pyelonephritis
Any location	Abdominal wall: herpes zoster, muscle strain, hernia Other: bowel obstruction, mesenteric ischemia, peritonitis, narcotic withdrawal, sickle cell crisis, porphyria, IBD, heavy metal poisoning

IBD = inflammatory bowel disease; IBS = irritable bowel syndrome; PID = pelvic inflammatory disease.

^1^Cartwright SL, Knudson MP: **Evaluation of acute abdominal pain in adults**. *Am Fam Physician* 2008, **77**:971-8. Review.

Associated symptoms, medical history or drug therapy often allow the physician to further focus on the differential diagnosis. However a confident and accurate diagnosis can be made solely on the basis of medical history, physical examination, and laboratory test findings in only a small proportion of patients so that imaging plays a pivotal role [4].

Today, surely the integrated imaging, and in particular the use of multidetector Computed Tomography (MDCT) has revolutionised the clinical approach to this condition, simplyfing the diagnosis but burdening the radiologists with the problems related to the clinical management [5,6]. However although CT emerging as a modality of choice for evaluation of the acute abdomen, ultrasonography (US) remains the primary imaging technique in the majority of cases, especially in young and female patients, when the limitation of the radiation exposure should be mandatory, and often associated with conventional radiography, limiting the use of CT in cases of nondiagnostic US and in all cases where there is a discrepancy between the clinical symptoms and negative imaging at US. Another consideration is that both conventional radiography and US are widely available and easily accessible in the emergency department, even if their effectiveness in the diagnosis and management is strictly related to their limits and in particular to the experience and awareness of the radiologist.

In this review, we discuss the role of US in the diagnostic management of acute abdomen. Our focus is acute abdominal in general, but we also discuss a number of frequently urgent diagnoses according to the age and sex of patients.

## Main body

### 1. US Examination and Acute Abdominal Pain: general consideration

US is an imaging modality widely available in the Emergency Department. The lower cost and in particular the lack of radiation exposure are the most important advantages of US compared to CT. Furthermore US is a real-time dynamic examination and this characteristic conveys dynamic information about bowel motility, and changes in position and to depict blood flow. A variety of causes may impair peristalsis, including high-grade small-bowel obstruction, ischemia, enteritis, and infiltrative processes. Another important advantage of US examination is the possibility to correlate the US findings with the point of maximal tenderness. The most common US technique used to examine patients with acute abdominal pain is the graded-compression procedure [7]. With this technique, interposing fat and bowel can be displaced or compressed by means of gradual compression to show underlying structures. Furthermore, if the bowel cannot be compressed, the noncompressibility itself is an indication of pathology (inflammation such as appendicitis, intussusception, malignancy or luminal distension resulting from obstruction) [4]. Another example of dynamic examination is the evaluation of bowel hernias, mesentery, and omentum through the Valsalva manoeuvre. This manoeuvre may reveal an intermittent hernia, may show the contiguity of a mass with the intraperitoneal space, allowing better depiction of the hernia sac or abdominal wall defect, and showing reducibility [8]. Curved (3.5–5.0-MHz) and linear (5.0 –12.0-MHz) transducers are most commonly used, with frequencies depending on the application and the patient’s stature, on the depth of the anatomical structures and on the aim of the study, for example high-frequency linear transducers are most appropriate for the evaluation of hernias [9,10].

Colour and power Doppler imaging supplement the information provided by gray-scale imaging, with increased vascularity visualised in a number of inflammatory, infectious or neoplastic diseases. For example hyperemia, both of the bowel wall and adjacent mesentery, is a marker of disease activity in inflammatory bowel disease [11] whereas diminished vascularity is a specific, although probably not sensitive, sign of ischemia [12-14]. In experienced hands, ultrasound may be as effective as radiography at diagnosing pneumoperitoneum [15]

Finally transvaginal imaging could be very useful in the evaluation of abdominal pain from gynaechologic causes and it could also be useful for the evaluation of deeply positioned appendixes, terminal ileitis, sigmoid or rectal inflammation [16].

US can provide useful information for about 56% of patients with acute abdominal pain, and as reported by several authors who claim that US is considerably helpful in making the correct diagnosis, in the initial evaluation of the patients with acute abdominal pain [17]. Since 1991, McGrath et al [18] reported that US either yielded unique diagnostic information or confirmed one of the differential diagnoses in 65% of patients. In a more recent study [19] among 300 patients who presented with acute abdominal pain the US revealed a different diagnosis than the clinical impression in 69 (23%; 95%CI, 18.2-27.7%), and confirmed the diagnosis in 121 (40%; 95%CI, 34.4-45.5%) patients. The US changed the treatment plans in 47% (95%CI, 41.3-52.6%) of the patients. When US results were compared with the discharge diagnosis, there was concordance in 238 (79.3%; 95%CI, 74.3-83.6%) patients but not in 62 (20.6%; 95%CI, 16-25.1%). Among 121 patients the initial clinical impression agreed with the US diagnosis and there was concordance with the discharge diagnosis in 105 (86.7%; 95%CI, 80-92.7%). The concordance of US findings with the discharge diagnosis was significantly higher than that of the initial clinical impression statistically.

#### 1.1 US in Paediatric Acute Abdomen

**Figure 1:**
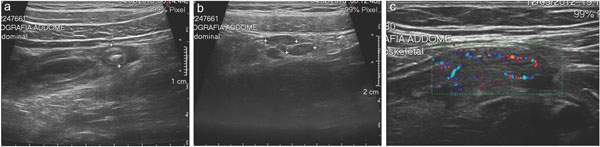
Appendicytis: US findings. (a) enlarged (>6 mm) and thickened appendix with appendicolitis and a rim of periappendiceal fluid; (b) enlarged lymphonodes along the ileo-colic vessels and (c) signs of hyperemia at Color imaging.

Acute abdomen is a commonly presented complaint in paediatric emergency. The differential diagnosis of acute abdomen in children varies according to the age, these are reported in Table 2 [20]. The pain is often due to a wide range of mild self-limiting medical causes, but it sometimes may be due to an acute potentially life threatening, surgical/medical illness (8%), so that the goal of emergency management is to ensure that life-threatening surgical causes are not missed [20]. These include appendicitis, non -reducibile intussusception, intestinal obstruction, incarcerated hernia, volvulus, ovarian/testicular torsion, perforated viscus with diffuse peritonitis, rupture tumor. CT and US have been less well evaluated in children than in adults, but there is increasing data on imaging use in the paediatric population. Several factors are unique in children, including increased radiosensitivity to ionizing radiation and smaller body size and less body fat, favouring the initial use of US [20]. Even if procedural imaging depends on clinical manifestation and clinical suspicion of the etiology of acute abdomen, US abdomen examination is the first investigation in almost all cases with moderate and severe abdominal pain. If appendicytis (14% of the causes of acute abdomen at the Emergency Department) is suggested from a clinical examination, US is the imaging procedure of choice with a sensitivity of 85 % to 90 % in the hands of an experienced radiologist, allowing the diagnosis without radiation exposure [21,23]. An aperistaltic, enlarged appendix (>6 mm) which is non compressible and with a rim of periappendiceal fluid is highly suggestive of appendicitis (Fig. 1). The visualisation of appendicolitis has been shown to have a low positive predictive value for the diagnosis of appendicitis because these may also be present in individuals who do not have appendicitis. US is often necessary to confirm the diagnosis of appendicitis because, despite having high sensitivity (upto 100%), the clinical evaluation has relatively low specificity (73%) [24] and a false positive diagnosis may lead to unnecessary surgical exploration, which is associated with increased mortality risk, prolonged hospital stay, and an increased infection-related complication risk [25]. On the contrary a false-negative (missed) diagnosis can lead to prolonged time to treatment and an increased risk of perforation [26], that is very high in the younger group at the time of diagnosis (almost 100% below 1 y, and 60-65% below 6 y of age).

**Table 2:** Causes of abdominal pain according to age of child ^2^

Birth to 1 year	2-5 years	6-11 years	12-18 years
*Medical*			
Infantile colic	Gastroenteritis	Gastroenteritis Constipation	Gastroenteritis Constipation
Gastroenteritis	Lower Lobe Pneumonia	Abdominal Tuberculosis	Lower Lobe Pneumonia
Constipation	Constipation	Bowel disease	Pharyngitis
Urinary Tract Infection	Urinary Tract Infection	Functional Pain	Dysmenorrhea
	Sickle Cell Crisis	Lower Lobe Pneumonia	Mittelschmerz
	Henoch-Schonlein purpura	Pharyngitis	Pelvic Inflammatory Disease
	Mesenteric Lymphadenitis	Urinary Tract Infection	Inflammatory Bowel Disease
		Pneumonia	
		Sickle Cell Crisis	
		Henoch-Schonlein purpura	
		Mesenteric Lymphadenitis.	
*Surgical*			
Intussusception	Appendicitis	Appendicitis	Appendicitis
Volvulus/malrotations	Intussusception	Cholecystitis	Ectopic Pregnancy
Incarcerated Hernias	Volvulus	Testicular Torsion	Testicular Torsion
Hirschsprung’s disease	Trauma	Trauma	Ovarian Torsion
Necrotizing Enterocolitis			

^2^ Balachandran B, Singhi S, Lal S: **Emergency management of acute abdomen in children**. *Indian J Pediatr* 2013, **80**:226-34.

**Figure 2:**
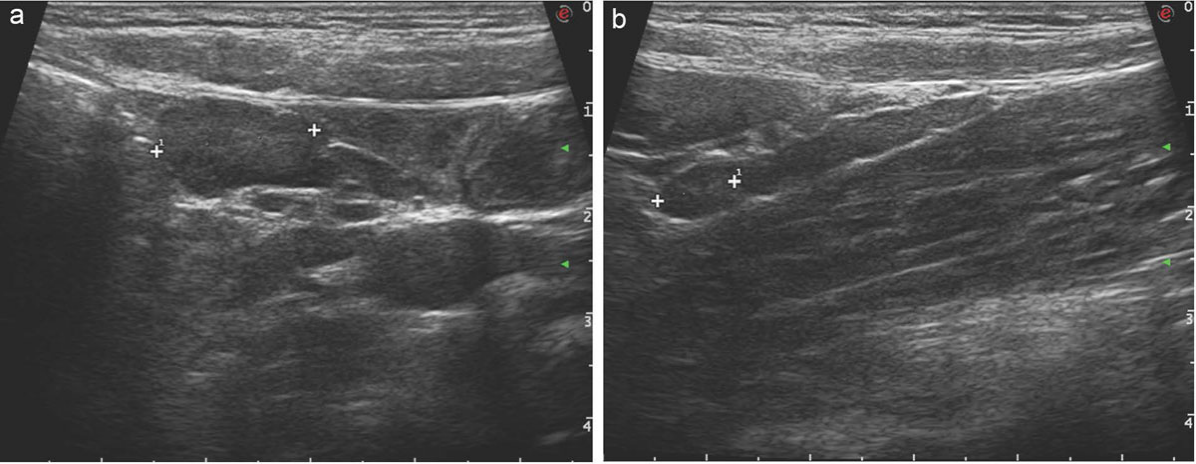
Adenomesenteritis: US findings. multiple enlarged lymphnodes (a), some of these realising a chain in the mesentery.

US is also a sensitive tool for adenomesenteritis (Fig. 2) and bowel obstruction (25% of the causes of acute abdomen at the ED) with a reported accuracy for the latter of about 81% [27]. Fluid-filled loops are easily visualised at US, and one can easily differentiate between a mechanical obstruction and paralytic ileus by visualising peristaltic movement [27]. Furthermore the altered relationship of superior mesenteric artery and vein is an important clue to malrotation. However US has important limitations: gas-filled loops may obscure the underlying abnormality, which have important treatment management-related implications, and the obstruction is difficult to stage accurately. The ACR claims that US is the least appropriate imaging modality when high or low-grade SBO is suspected [3]. US study is the examination of choice in the paediatric population also in cases of intussusception, that is typically seen in a previously well infant who had an episode of acute diarrhoea but that may also be seen in older children upto 5-6 y of age in presence of a pathologic “lead point” for intussusception, such as polyps, lymphoma, Meckel’s diverticulum, or Henoch-Schonlein purpura. US findings in intussusceptions include the target sign, a single hypoechoic ring with a hyperechoic centre and the “pseudokidney” sign, superimposed hypo- and hyperechoic areas representing the edematous walls of the intussusceptum and layers of compressed mucosa. Doppler flow may be used to identify complications like bowel ischemia. If signs of intussusception are not identified by US, proceeding with a barium or air enema should still be considered, if the clinical suspicion is high [20]. Alternatively hepatobiliary disease and acute pancreatitis are easily confirmed by US and liver/pancreatic function tests. US represents the investigation of choice in emergency also for suspected cases of genitourinary conditions (such as ovarian torsion, ruptured ovarian cyst, ectopic pregnancy, and testicular torsion), and renal calculi.

Overall US examination of abdomen was found to be diagnostic in 48 % of acute abdomen in the paediatric population and supportive in further 18.6 %. Even if CT should be more accurate, US is nearly as good in experienced hands and, given the lack of ionizing radiation, is the preferred examination in children, particularly if equivocal results are followed up by CT [28]. Thus, the CT-after-ultrasound approach seems to have excellent accuracy, with reported sensitivity and specificity of 94% [29,30].

#### 1.1 US in Adult Acute Abdomen

##### - Male

**Figure 3:**
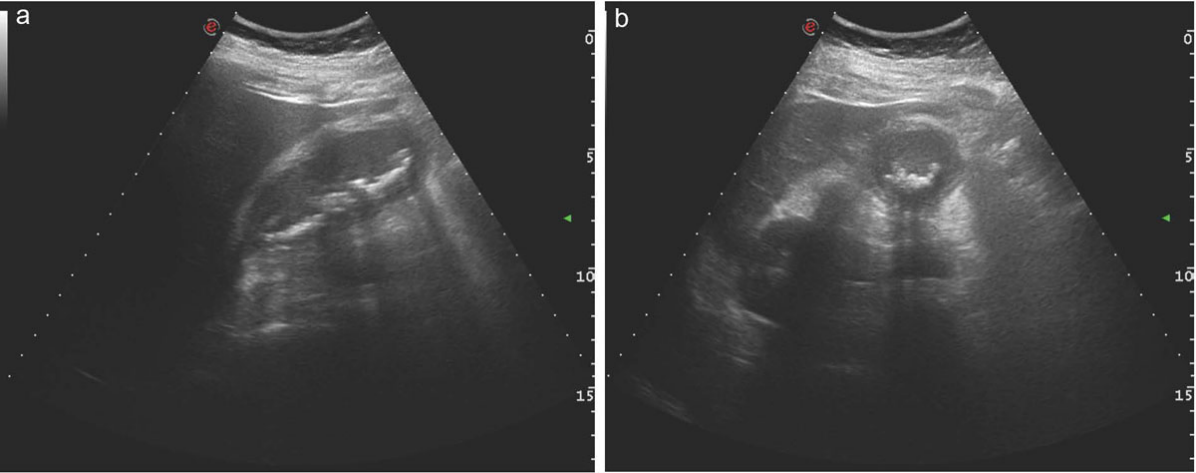
Cholecystitis: US findings. multiple gallstones associated with gallbladder wall thickened are depicted in both longitudinal (a) and axial (b) images.

**Figure 4:**
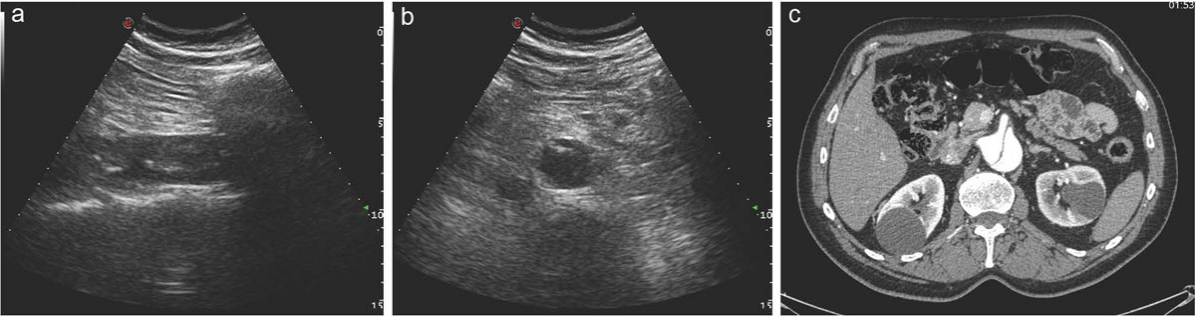
Aortic dissection identifyed at US examination (a and b) and confirmed at contrast-enhanced CT examination (c) at the Emergency Department in a 65y old patient suffering from acute abdomen.

In the adult population the goal of emergency management is the same of that in the paediatric population: to ensure that life-threatening surgical causes are not missed. In fact although most abdominal pain is benign also in the adult population, as many as 10% of patients in the emergency department setting and a lesser percentage in the out-patient setting have a severe or life-threatening cause or require surgery [2]. Acute appendicitis, diverticulitis, cholecystitis, and bowel obstruction are common causes of acute abdominal pain, but other important, even if less frequent conditions, that may cause acute abdominal pain include perforated viscus or vascular diseases such as aortic dissection and mesenteric ischemia [2]. Pathologies of the abdominal wall, such as herpes zoster, should also be considered because they are often misdiagnosed. Although location of abdominal pain guides the clinical suspicion, associated signs and symptoms are predictive of certain causes of abdominal pain and can suggest a differential diagnosis. According to the ACR criteria recommendations, initial imaging studies are based on the location of abdominal pain: US is recommended when a patient presents with right upper quadrant pain (US should be considered the primary imaging technique, for example for patients clinically suspected of having acute cholecystitis, Fig. 3), Computed tomography (CT) with intravenous contrast media is recommended for evaluating adults with acute right lower quadrant pain (CT is considered to be better than US for diagnosing appendicitis and can detect extracolonic causes of abdominal pain in adult population); CT is also recommended for patients with left lower quadrant pain (sigmoid diverticulitis is the most common cause of left lower quadrant pain in adults, and CT has a reported sensitivity of 79 to 99% for detecting the condition) [31-33]. Left upper quadrant pain is caused by a variety of clinical conditions; therefore, imaging recommendations are not clear-cut. If the patient’s history and physical examination suggest esophageal or gastric pathology, endoscopy is recommended. In other patients with left upper quadrant pain, CT is useful because it provides imaging of the pancreas, spleen, kidneys, intestines, and vasculature[34,35,36]. In general, CT is highly effective at identifying patients with nonspecific abdominal pain who need urgent intervention (LR+ = 9.20, LR– = 0.09) [37]. However, although the ACR criteria limiting the current role of ultrasound for adult acute abdomen evaluation with CT is emerging as the primary modality of choice for this condition, mounting concerns regarding diagnostic radiation and health care costs could affect the clinical management and shift the utilisation back towards US, which is widely available and easily accessible in the ED. As reported above US has the advantage of a real-time dynamic examination and this characteristic conveys dynamic information about bowel motility and depicted blood flow, suggesting a variety of pathologies, including perforated viscus. A perforation can be in fact diagnosed at US when echogenic lines or spots with comet-tail reverberation artefacts representing free intraperitoneal air are seen adjacent to the abdominal wall in a supine patient. A sensitivity of 92% and a specificity of 53% have been reported for the detection of perforation with US and constitute an overall accuracy of 88% [38]. It is important to note that establishing the cause and location of the perforation is difficult with US. Overall, US is useful to identify patients that need a possible urgent surgical approach, for example patients suffering from an aortic dissection (Fig. 4) or and abdominal aneurism rupture, to obtain a quick and less invasive treatment [39], but also this fast-time execution and wide diffusion allows the facilitation of second-level-imaging, essential to direct the correct management of acute abdominal pain in patients of different ages [3].

##### - Female

**Figure 5:**
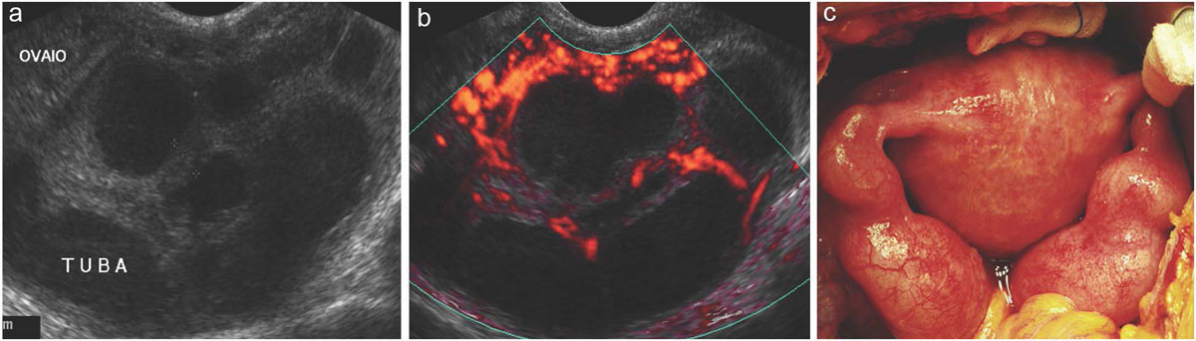
Gray-scale US examination (a) and Colour imaging (b) clearly depict a pelvic inflammatory disease confirmed at surgery. (Courtesy of Prof. F.M. Severi, University of Siena).

The female gender represents a special population from a diagnostic point of view in the clinical suspicion of acute abdomen because abdominal pain in women may be related to pathology in the pelvic organs. Ectopic pregnancy, pelvic inflammatory disease, and hemorrhagic ovarian cysts are the most commonly diagnosed gynaecologic conditions presenting with acute pelvic pain. Ovarian torsion and degenerating fibroids occur less frequently. Other causes to consider include endometriosis, and postpartum causes such as endometritis, or ovarian vein thrombosis. Finally, nongynaecologic conditions may overlap in their presentation of acute pelvic pain and should also be considered; the most important of these is acute appendicitis [1]. US is the primary and sometimes the only necessary imaging tool in the assessment of acute pelvic pain in women. The true value of ultrasound in female acute abdominal pain lies in it’s ability to detect gynaecologic disorders and effectively rule out other causes of acute abdominal pain that require surgical repair. If a gyneacologic disorder is confirmed, other imaging studies might be unnecessary, thereby reducing cost, length of hospitaliztion, and adverse complications of CT (contrast material reactions, and radiation exposure) [40]. In the nonpregnant patient, US early diagnosis and treatment of ovarian torsion can preserve ovarian function. US also has both diagnostic and therapeutic capabilities in patients with pelvic inflammatory disease through guidance of abscess drainage via the transvaginal route, Fig. 5 [41].

**Figure 6:**
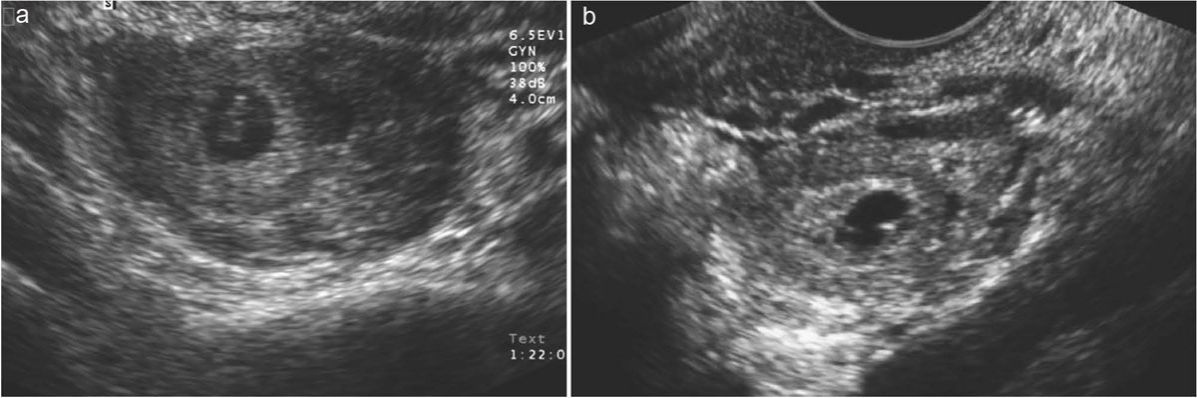
Gray-scale US examination (a and b) shows two ectopic ovarian pregnancy demonstrating a well-defined gestational sac.

In women of reproductive age, special attention to pregnancy, including ectopic pregnancy, and loss of pregnancy is critical in forming an appropriate differential diagnosis[42,43]. The possibility of pregnancy modifies the likelihood of disease and significantly changes the diagnostic approach (e.g., avoidance of radiation exposure in diagnostic testing). In these patients, and in particular, in suspected ectopic pregnancy, US, together with quantitative measurements of hCG levels, can be considered the best imaging procedure to guide the diagnosis and in determining the size and location of the ectopic pregnancy, and presence of bleeding, which in turn helps guide treatment decisions (Fig. 6). MRI is the preferred test after inconclusive US findings in gynaecologic disorders whereas CT is more valuable for assessing nongynaecologic disorders or post-partum and post-operative infections [44].

##### - Elderly people

Older patients with acute abdomen present a particular diagnostic challenge because disease frequency and severity may be exaggerated in this population (e.g., a higher incidence of diverticular disease or sepsis in those with urinary tract infection) or on the contrary a reduction in symptom severity causing a misdiagnosis could be present. Furthermore, clinical presentation may differ significantly in older patients[45]. There are several diseases that should be considered in all older patients with abdominal pain because of the increased incidence and high risk of morbidity and mortality in these patients. Occult urinary tract infection, perforated viscus, and ischemic bowel disease are potentially fatal conditions commonly missed or diagnosed late in older patients[46,47]. Another important topic in elderly patients is aortic occlusion and Leriche syndrome [48,49]. US imaging is the first step in these patients, in particular if the clinical suspicion suggests a major vascular etiology, such as aortic occlusion or rupture, but due to the reduced risk exposure dose, the imaging of choice is CT, also because more frequent pathologies are confirmed as bowel ischemia [50-53]. Finally another special population, from a clinical point of view, are the oncologic patients [54]. In addition to the commonly encountered acute conditions found in the general population, the oncologic patient is at increased risk of developing an acute condition due to local effects of the primary tumour and metastases, sequaelae of treatment (in particular with the new therapeutic agents) and altered immune response. Also in these patients US imaging should be used in the first step of the diagnostic approach, especially if the patient is young and with curable tumours (like lymphoma) but CT remains the imaging of choice in case of undeterminate US.

## Conclusion

Given its availability, relatively low cost, and absence of ionizing radiation or need for contrast materials, US has maintained an important role in the evaluation of the acute abdomen even during the recent diffusion of MDCT. Awareness of normal and pathologic sonographic appearances of bowel and attention to technique will enable radiologists to make optimal use of this imaging modality.

## Competing interests

The authors declare that they have no competing interests.

## Declarations

This article has been published as part of *Critical Ultrasound Journal* Volume 5 Supplement 1, 2013: Topics in emergency abdominal ultrasonography. The full contents of the supplement are available online at http://www.criticalultrasoundjournal.com/supplements/5/S1. Publication of this supplement has been funded by the University of Molise, University of Siena, University of Cagliari, University of Ferrara and University of Turin.
